# Experimental verification of Advanced Collapsed‐cone Engine for use with a multichannel vaginal cylinder applicator

**DOI:** 10.1002/acm2.12061

**Published:** 2017-03-20

**Authors:** Brie Cawston‐Grant, Hali Morrison, Geetha Menon, Ron S Sloboda

**Affiliations:** ^1^ Department of Medical Physics Cross Cancer Institute Edmonton AB T6G 1Z2 Canada; ^2^ Department of Oncology University of Alberta Edmonton AB T6G 2R3 Canada

**Keywords:** ACE, brachytherapy, gynecology, phantoms, radiochromic film

## Abstract

Model‐based dose calculation algorithms have recently been incorporated into brachytherapy treatment planning systems, and their introduction requires critical evaluation before clinical implementation. Here, we present an experimental evaluation of Oncentra^®^ Brachy Advanced Collapsed‐cone Engine (ACE) for a multichannel vaginal cylinder (MCVC) applicator using radiochromic film. A uniform dose of 500 cGy was specified to the surface of the MCVC using the TG‐43 dose formalism under two conditions: (a) with only the central channel loaded or (b) only the peripheral channels loaded. Film measurements were made at the applicator surface and compared to the doses calculated using TG‐43, standard accuracy ACE (sACE), and high accuracy ACE (hACE). When the central channel of the applicator was used, the film measurements showed a dose increase of (11 ± 8)% (k = 2) above the two outer grooves on the applicator surface. This increase in dose was confirmed with the hACE calculations, but was not confirmed with the sACE calculations at the applicator surface. When the peripheral channels were used, a periodic azimuthal variation in measured dose was observed around the applicator. The sACE and hACE calculations confirmed this variation and agreed within 1% of each other at the applicator surface. Additionally for the film measurements with the central channel used, a baseline dose variation of (10 ± 4)% (k = 2) of the mean dose was observed azimuthally around the applicator surface, which can be explained by offset source positioning in the central channel.

## 1 Introduction

The standard treatment planning process for brachytherapy involves use of the well‐accepted TG‐43 dose calculation formalism,^1^ which does not account for material heterogeneities. This is in contrast with current treatment planning systems (TPSs) used in external beam radiation therapy (EBRT), which account for the effects that heterogeneities have on dose. However, recent developments have led to two commercial TPSs that include the option of model‐based dose calculation algorithms (MBDCAs) for high dose rate (HDR) Ir‐192 brachytherapy: Oncentra^®^ Brachy (OcB; Elekta, Stockholm, Sweden), which utilizes the Advanced Collapsed‐cone Engine (ACE),^2^, ^3^, ^4^ and BrachyVision (Varian, Palo Alto, CA, USA), which uses the grid based Boltzmann solver Acuros™.^5^

For gynecological cancer treatments, the present clinical situation that assumes all materials are composed of water has the potential to introduce errors in dose calculations.^6^, ^7^, ^8^, ^9^, ^10^ These errors can potentially cause an undesired increase in radiation dose delivered to organs at risk (OARs: rectum, bladder, and sigmoid colon) or reduced dose to the tumor. In clinical practice it is common for OAR doses to approach their upper limit while trying to achieve acceptable target coverage. Consequently, calculating OAR doses accurately might be the difference between successful OAR sparing and a radiation‐related OAR complication. Furthermore, neglecting attenuation when using applicators with OAR shielding has been shown to overestimate D_2cc_ rectum and D_2cc_ bladder by 6.2% and 3.4%, respectively.^8^ In addition, Hyer et al. reported that Acuros™ yielded a dose difference of approximately 2% relative to TG‐43 when both tissue and applicator heterogeneities were considered based on planning CT images.^9^

The prospect of bringing brachytherapy dose calculation accuracy up to the level of that of EBRT is very appealing. However, improved dose calculation accuracy does not necessarily equate to clinical benefits, and also requires longer calculation times. Therefore, it is important to critically evaluate and commission the MBDCAs both computationally and experimentally until their performance and contribution is established for a given treatment procedure.^11^ As stated above, the use of MDBCAs has been investigated for some gynecological applicator models; e.g., tandem‐ovoid^6^, ^7^, ^8^, ^9^ and tandem‐ring applicators.^10^ However, the performance of MDBCAs has not yet been reported for a multichannel vaginal cylinder (MCVC). Therefore, we present an experimental evaluation of OcB ACE v4.5 for a MCVC applicator using radiochromic film measurements. This evaluation focuses on ACE's ability to predict dose variations at the applicator surface caused by applicator‐based heterogeneities. Differences between ACE calculated doses and the current clinical standard (TG‐43) are used to evaluate the clinical implications of using ACE to predict heterogeneity‐induced dose variances. The thorough and valid evaluation of ACE required confirmation that the expected MCVC dimensions corresponded to the physical MCVC applicator, which was performed using micro‐CT imaging.

## 2 Materials and methods

### 2.A Film, applicator, and Oncentra^®^ Brachy

All film measurements were performed using Gafchromic™ EBT3 film (Ashland Specialty Ingredients, Wayne, NJ, lot #03181303 and #04201501). EBT3 has a 28 μm thick active layer of lithium pentacosa‐10,12‐dyinoic acid, and has a total thickness of 0.27 mm.^12^ The film calibration curves were determined using a 6 MV beam from a Varian Clinac^®^ iX‐S linear accelerator (linac) (serial #975, Varian Medical Systems, Palo Alto, CA). Analysis of the images was performed in MATLAB v7.11 (MathWorks, Natick, MA,USA).

**Figure 1:**
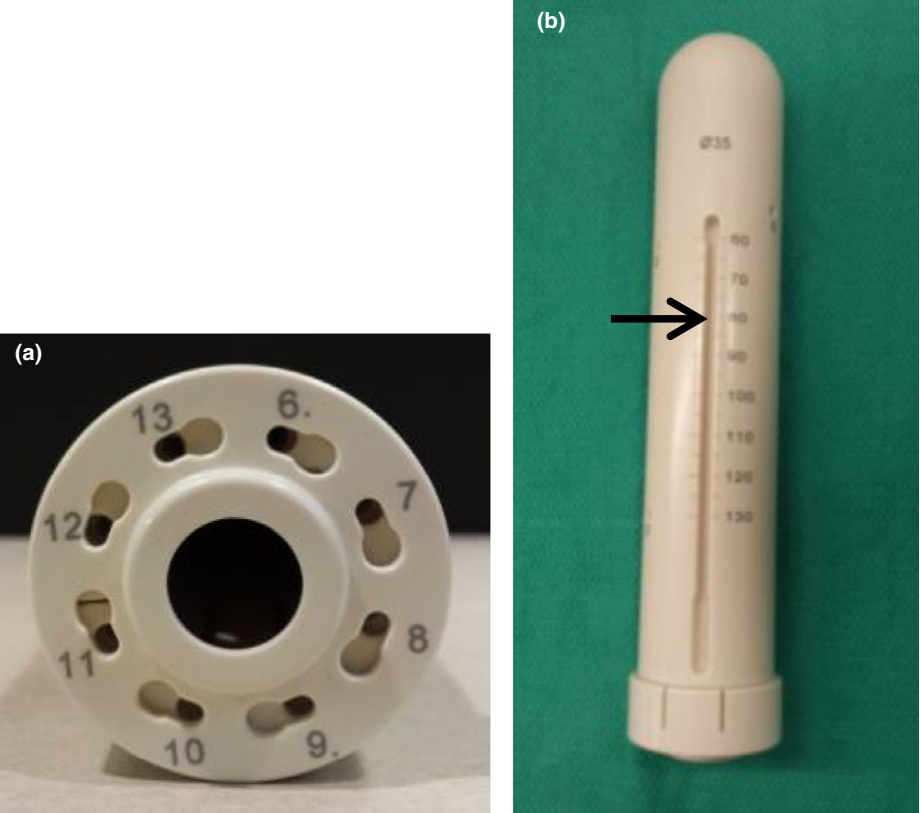
An axial view of the multichannel vaginal cylinder (a), a full view of the MCVC with an outer groove visible (identified with an arrow, (b)).

Film measurements were made at the surface of a 35 mm diameter Vaginal CT/MR MCVC (Elekta, Stockholm, Sweden, part #110.761) with a vaginal tube (part #101.002), which is shown in Figs. 1(a) and 1(b). It has eight peripheral channels, and a vaginal tube may be inserted into the MCVC to provide a central channel. There are two 5 mm deep grooves on the outside of the applicator where a perineal bar can be attached to provide fixation (Fig. 1(b)). The grooves begin 60 mm from the tip of the applicator. The majority of the applicator is composed of polyphenylsulfone (PPSU) plastic, which has a mass density of 1.29 g/cc.

TG‐43 and ACE dose calculations were compared for the 35 mm MCVC model with the vaginal tube in the OcB Applicator Modeling module of OcB v4.5 (research version) (Elekta, Stockholm, Sweden). The Applicator Modeling module in OcB offers a standardized description of the 3D geometry, applicator materials, and source path for a library of brachytherapy applicators. For non‐ring applicators, the source path is modeled to be along the center of the catheter lumen. Details of how ACE calculates dose are reported in a recent article by Ahnesjö et al.^4^ A few of the key principles will be described here. ACE calculates dose as the sum of contributions from primary photons, once‐scattered photons, and any residual scattering. The primary dose is calculated using a ray trace of the primary photons in a grid, which generates scatter energy that is input into the collapsed cone superposition convolution (CCSC) algorithm. CCSC utilizes angular discretization of radiation transport directions and pre‐calculated dose deposition point kernels in water, scaled to reflect the influence of inhomogeneities.^13^ The transport directions are a uniform spherical tessellation around each scattering center.^14^, ^15^, ^16^ In ACE, high (hACE) and standard (sACE) accuracy calculation modes use different numbers of transport directions for first scatter and residual scatter components.^4^, ^16^ When a single dwell position is used, hACE calculates the scatter dose using 1620/320 first/residual scatter transport directions, and sACE uses 320/180 first/residual scatter transport directions. The number of transport directions decreases as the number of dwell positions increases. To speed up the calculations, multiple calculation grid resolutions are used. The white paper from Elekta^2^ defines the grid resolution in terms of a voxel size, however, it will be referred to as the “grid size” in this paper. The grid sizes used in ACE calculations depend on the distance from each dwell position. To determine the grid size, ACE first defines a box that contains all the dwell positions. A margin is then added to that box, which contains a particular grid size. For example, hACE has a 1 mm grid size up to 80 mm from a dwell position in the x, y, and z directions, when more than one dwell position is used. Table 1 gives the margins and corresponding grid sizes for the hACE and sACE calculations, which also apply to the primary dose ray trace.

**Table 1:** Grid sizes for the high and standard accuracy ACE calculations when the number of dwell positions is between 2 and 50

Margin (mm)	High accuracy grid size (mm)	Standard accuracy grid size (mm)
10	N/A	1
80	1	2
200	2	5

### 2.B Radiochromic film measurements

#### 2.B.1 Calibration of film

The radiochromic film calibration curves were determined using a 6 MV linac beam for each film batch. EBT3 film has been shown to be energy independent for photons with energy greater than 50 keV, therefore, the use of the 6 MV beam for calibration purposes is valid.^17^, ^18^ Pieces of film, 4 × 4 cm^2^ in size, were irradiated at 13 dose levels between 0 and 10 Gy. The linac output was measured with an ion chamber (Capintec PR‐06, Ramsey, NJ, USA) before and after the film irradiations, and the intended dose levels were corrected with a multiplicative factor equal to the measured output divided by the expected output. The irradiations were performed in a 10 × 10 cm^2^ beam at 100 cm source‐to‐axis distance within 20 × 20 cm^2^ slabs of solid water. The films were irradiated at the depth of maximum dose by placing a build‐up of 1.5 cm of solid water above the film; 12 cm of solid water was placed below the film for backscatter. The film was scanned with an Epson Expression 10000 XL scanner (Seiko Epson Corp., Nagano, Japan) at 72 dpi, 48‐bit RBG color (16‐bit per color), and the scans were saved as tagged image file format images. For each color channel, fitting parameters a, b, and c in Eq. (1) were determined by the non‐linear least squares effective variance method solved by direct optimization as described by Ramos and Azorin.^19^ In Eq. (1), X(D) is the normalized film response in relative pixel values (PVs) (measured PV divided by maximum PV 65535) and D is the dose in cGy. The optical density (OD) was then determined by Eq. (2).

(1) $$X\left(D\right)=\frac{a+bD}{c+D}$$

(2) $$OD=-\log_{10}\left(\frac{a+bD}{c+D}\right)$$

To perform measurements of unknown doses, triple channel analysis was used, wherein the dose determined from each channel was corrected for film thickness heterogeneities using an iterative approach. Uncertainties in the film measurements follow from the uncertainty principles outlined in the NIST Technical Note 1297,^20^ and implemented by Morrison et al.^21^ and Chiu‐Tsao et al.^22^ The final dose was taken as a weighted mean using the relative uncertainties associated with each channel. Details pertaining to the uncertainty calculations are described in the Appendix.

#### 2.B.2 Measurements with multichannel vaginal cylinder applicator

Using the MCVC model available in the Applicator Modeling module of the OcB software, a dose of 500 cGy was specified to a set of dose points on the surface of the applicator, over a 70 mm length. To isolate dosimetric effects caused by heterogeneities, it was desirable to specify as uniform a dose as possible to the surface of the applicator. Along the length of the applicator, prescription points were placed every 5 mm between 60 mm and 130 mm from the applicator tip, and at every 22.5 degrees around the circumference of the applicator, for a total of 240 points. Two different sets of dwell positions were used: positions activated only in the central channel, or only in the peripheral channels of the applicator. When the central channel was loaded, 20 dwell positions spaced 5 mm apart were activated, with the first dwell position 50 mm from the applicator tip. When the peripheral channels were loaded, 136 dwell positions (17 per channel) spaced 5 mm apart were activated, with the first dwell position 54 mm from the applicator tip. Dose was geometrically optimized on the surface of the applicator.

**Figure 2:**
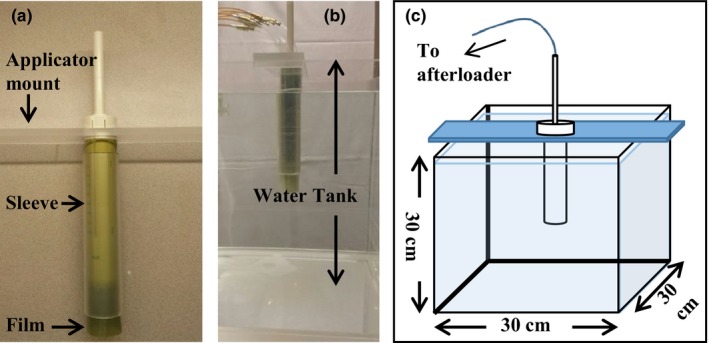
The multichannel vaginal cylinder with Gafchromic™ film wrapped around the exterior and held in place by an acrylic sleeve (a). The applicator with film placed in the 30 × 30 × 30 cm^3^ water tank (b). A schematic of the 30 × 30 × 30 cm^3^ water tank (c).

Film measurements were made at the surface of the MCVC applicator by wrapping a 20 cm long film piece around the outside of the applicator. The film was held in place with a 3 mm thick acrylic cylindrical sleeve whose inner radius was 0.5 mm larger than the applicator. Irradiations using the MCVC applicator were performed in a 30 × 30 × 30 cm^3^ water tank with the applicator oriented vertically (Fig. 2). The dose was delivered to the film under two conditions: the applicator was either placed in the water prior to being inserted into the film and sleeve to ensure the applicator grooves were filled with water, which will be referred to as the “water‐in‐grooves” set‐up in this paper, or the film and sleeve were applied to the applicator prior to insertion into the water and a tight waterproof latex sleeve was placed around the applicator to ensure water did not enter the grooves, which will be referred to as the “air‐in‐grooves” set‐up.

For dose values obtained from the film measurements, an inverse square (IS) correction was applied to account for the thickness of the film, which relocates the measurements to the surface of the applicator. When the central channel was used, the IS correction equated to (17.635/17.5)^2^ = 1.0155. When the peripheral channels were used, the IS correction for a point on the surface of the applicator was determined by a weighted average of the individual IS corrections from all dwell positions. IS corrections were calculated for 16 points at different azimuthal angles on the surface of the applicator. The average IS correction was 1.0087, which was applied to the peripheral channel film measurements. Dose profiles were then taken across the film to investigate the variation in dose around the surface of the applicator. The dose profiles were averaged over a height of 100 pixels (35.3 mm centered on the prescribed dose region) and obtained using a moving average width of 5 pixels (1.8 mm) with respect to the dose maps. The horizontal moving average was performed to account for the film possibly being slightly tilted during irradiation or scanning. An average dose was calculated from the same area of the film used to obtain the averaged profiles. When an average dose was calculated, the associated type A uncertainty was added in quadrature with the uncertainty arising from use of the derived calibration curve.^21^, ^22^ All uncertainties are stated with a coverage factor of 2 (k = 2). The averaged dose profiles were also used to obtain values for dose variations across the surface of the applicator: the maximum dose variation was calculated as the difference between the minimum and maximum doses; the size of the largest peak refers to the height of the dose difference observed at the surface of the two grooves in the applicator; and the baseline variation is the difference between the minimum and maximum doses at locations other than the peaks at the grooves or peripheral channels of the MCVC.

### 2.C Micro‐CT imaging

To fairly assess ACE using experimental film measurements, the MCVC applicator model in the OcB Applicator Modeling module must be verified against the physical MCVC applicator. Additionally, initial film measurements revealed a non‐uniform and slightly lower average dose at the applicator surface compared to TG‐43. To further investigate the origin of the dose variations and verify the applicator library model, micro‐CT (μCT) images were obtained of the MCVC applicator, using a Siemens Inveon™ Hybrid Micro‐PET/CT Scanner (Siemens, Knoxville, TN, USA). The images were used to accurately measure the dimensions and geometry of the applicator, film, and sleeve, which could contribute to deviations in the measured dose from the specified dose. The images had isotropic voxels with a side length of 31μm. Image analysis was performed using ImageJ v1.49 (National Institutes of Health, USA).

### 2.D TG‐43 and ACE dose calculations

The TG‐43 and ACE dose calculations were compared for the 35 mm MCVC applicator library model. The applicator was placed in a 30 × 30 × 30 cm^3^ virtual water box with a 34.8 mm diameter cylinder of air centered on the applicator to emulate the outer grooves filled with air. The cylinder of air was made to have a smaller diameter than the applicator to ensure the prescription dose points lay within the water and not air. ACE calculations were also performed with the outer grooves filled with water. Following the same procedure as in the experimental measurements, TG‐43 was used to specify a dose of 500 cGy to a set of dose points on the surface of the applicator over a 70 mm length. Two different sets of dwell positions were used: dwell positions activated only in the central channel, or only in the peripheral channels of the applicator. The activated dwell positions were the same as in the experimental plan. The dose was geometrically optimized on the surface of the applicator. The relative weightings of the dwell times when the central channel was loaded were identical to those used in the plan for the film measurement. When the peripheral channels were used, the doses to the prescription points on the surface of the applicator differed by an average of 0.04% from the doses to the same prescription points in the experimental plan. The dose was then recalculated using the sACE and hACE algorithms for the same dwell times. Additionally, with assistance from the vendor, we increased the innermost margin of the sACE calculation from 10 mm to 20 mm, which decreased the grid size at the applicator surface from 2 mm to 1 mm. The sACE calculation was repeated for the central channel with this increased margin, which will be referred to as the sACE‐20 mm calculation. To evaluate the clinical relevance of dose variations predicted by ACE, TG‐43 and ACE dose calculations were compared using point dose percent differences [Eqs. (3) and (4)]. In Eqs. (3) and (4), Δ*D*_*s*_ is the percent difference between the sACE calculated dose (*D*_*sACE*_) and the TG‐43 calculated dose (*D*_*TG43*_), and Δ*D*_*h*_ is the percent dose difference between the hACE calculated dose (*D*_*hACE*_) and *D*_*TG43*_.

(3) $$\Delta D_{s}\left(\%\right)=100\times \frac{D_{sACE}-D_{TG43}}{D_{TG43}}$$

(4) $$\Delta D_{h}\left(\%\right)=100\times \frac{D_{hACE}-D_{TG43}}{D_{TG43}}$$

The dose on the surface of the applicator was investigated with the same prescriptions points used to specify the dose in the experimental plans. The surface dose was averaged at nine locations along the length of the applicator for each angle, covering a span of 40 mm centered in the area that the dose was specified to. The uncertainty for individual dose points calculated using the TG‐43 formalism was taken to be 3.4%.^23^ The uncertainty associated with ACE calculated doses was estimated from analyses by others to be 5%.^24^, ^25^ When average doses were calculated from TG‐43 or ACE data, the associated type A uncertainty was added in quadrature with the 3.4% or 5% type B uncertainty, respectively. All uncertainties are stated with a coverage factor of 2 (k = 2). These uncertainties are further described in the Appendix. The averaged dose profiles were also used to obtain values for dose variations across the surface of the applicator: the maximum dose variation was calculated as the difference between the minimum and maximum doses; peak dose increases were calculated by subtracting the average dose for dose points not at peaks from the dose at the peak.

## 3 Results

## 3.A Radiochromic film measurements

**Figure 3:**
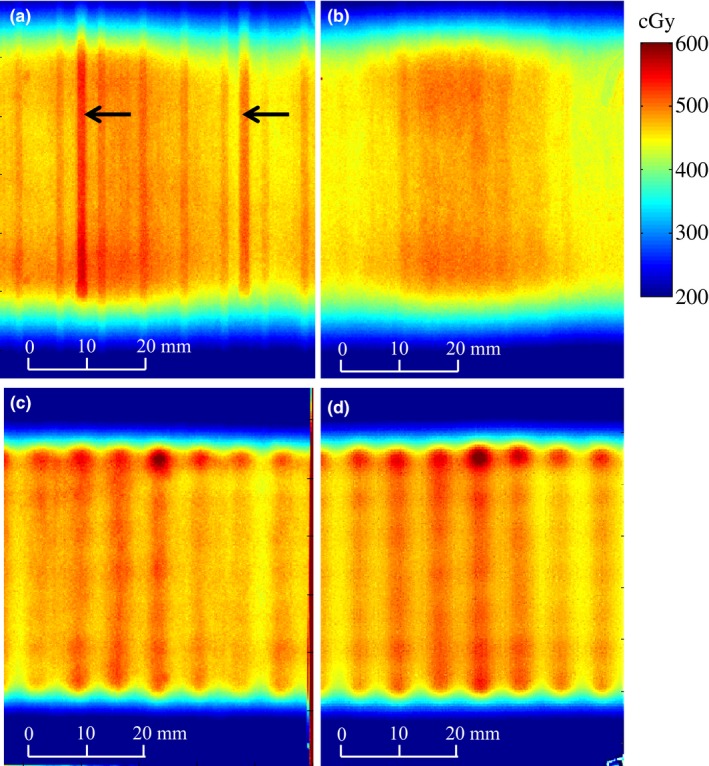
Film measurements taken at the surface of the MCVC with: (a) the central channel loaded and air‐in‐grooves (identified with arrows); (b) the central channel loaded and water‐in‐grooves; (c) the peripheral channels loaded and air‐in‐grooves; (d) the peripheral channels loaded and water‐in‐grooves.

Table 2 summarizes dose variations observed around the circumference of the applicator, which were obtained from the longitudinally averaged dose profiles taken across the film. The dose maps obtained from the film measurements are given in Fig. 3. The dose maps are oriented such that the base of the applicator (bottom of Fig. 1(b)) is at the top of the page. Figs. 3(a) and 3(b) show the measurements taken with the central channel of the MCVC applicator loaded. For the measurement in Fig. 3(b), the outer grooves were filled with water, and no distinct dose increases are visible above the grooves. In contrast, Fig. 3(a) shows the film measurements with air in the grooves, where an (11 ± 8)% increase in dose (i.e., the size of the largest peak) is seen just above the two grooves on the outside of the applicator. Fig. 3(c) and 3(d) show the film results for the irradiations performed using the peripheral channels of the MCVC, with air and water in the grooves, respectively. The maximum dose variation for the air‐in‐grooves setup was 3% less than the variation produced when the central channel was loaded (Fig. 3(a)). Additionally, the variation was more gradual. For the peripheral channel loading, the surface dose is very similar for the air‐in‐grooves and water‐in‐grooves set‐ups.

**Table 2:** Dose variations around the circumference of the MCVC applicator as measured with Gafchromic EBT3 film. The values are calculated from the longitudinally‐averaged dose profile data. Percentages are listed in parentheses beside the absolute doses and are relative to the mean dose. Expanded uncertainties (k = 2) are given

Channel(s)	Set‐up type	Maximum dose variation [cGy] (%)	Size of largest peak [cGy] (%)	Baseline variation [cGy] (%)	Mean dose [cGy]
Central	Air‐in‐grooves	77 ± 40 (16 ± 8)	54 ± 36 (11 ± 8)	37 ± 40 (8 ± 8)	477 ± 28
Central	Water‐in‐grooves	49 ± 18 (11 ± 4)	N/A	49 ± 18 (11 ± 4)	464 ± 16
Peripheral	Air‐in‐grooves	61 ± 38 (13 ± 8)	N/A	N/A	476 ± 30
Peripheral	Water‐in‐grooves	57 ± 26 (12 ± 6)	N/A	N/A	477 ± 14

All measurements with the MCVC having the central channel loaded showed baseline dose variations around the circumference of the applicator. This change in dose was on average (10 ± 4)% of the mean dose.

### 3.B Micro‐CT imaging

**Figure 4:**
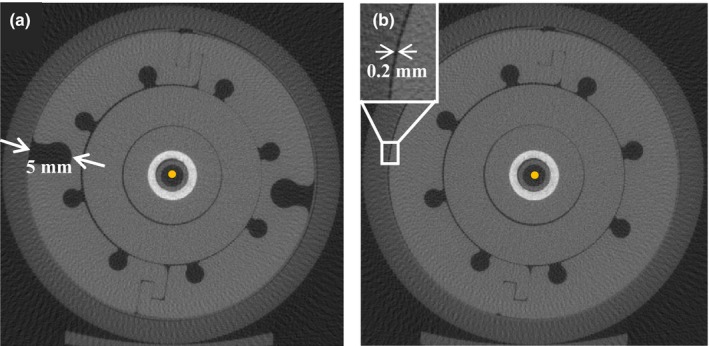
μCT cross‐sectional slices of the MCVC applicator with the vaginal tube inserted, and film wrapped around the outside and held in place with the acrylic sleeve. The central yellow dot is scaled to represent the microSelectron‐HDR^®^ Ir‐192 v2 source with a 0.9 mm diameter. The images reveal a groove depth of 5 mm (a), and a maximum air gap between the applicator and the film of 0.2 mm (b).

The μCT images of the MCVC applicator (Fig. 4) revealed a central channel diameter of (2.69 ± 0.04) mm, which is greater than the expected 2.5 mm given in the OcB applicator library model. The outer diameter of the applicator was measured to be (35.07 ± 0.04) mm. Additionally, the distance between the applicator and the film varied slightly around the circumference of the applicator and was 0.2 mm at its maximum.

### 3.C TG‐43 and ACE dose calculations

**Figure 5:**
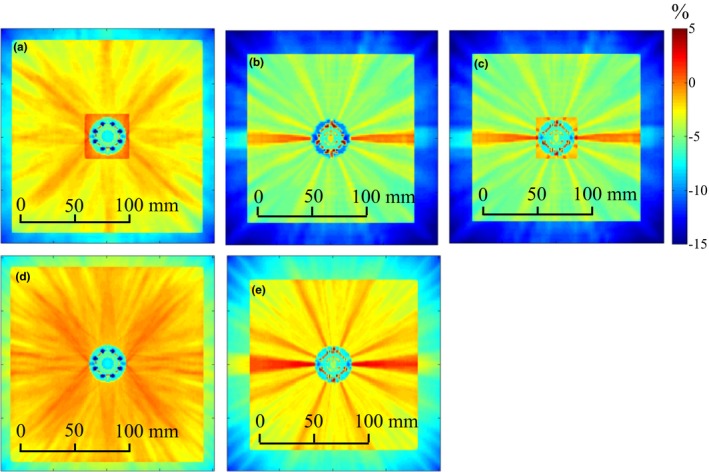
Percent dose differences between the sACE and TG‐43 dose calculations for a MCVC applicator having air in the surface grooves with the peripheral channels loaded (a), with the central channel loaded (b), and with the central channel loaded and the innermost margin increased from 10 mm to 20 mm (c). Percent dose differences between the hACE and TG‐43 dose calculations for the MCVC with the peripheral channels loaded (d) and with the central channel loaded (e). The surface grooves lie in the horizontal plane bisecting the applicator.

**Figure 6:**
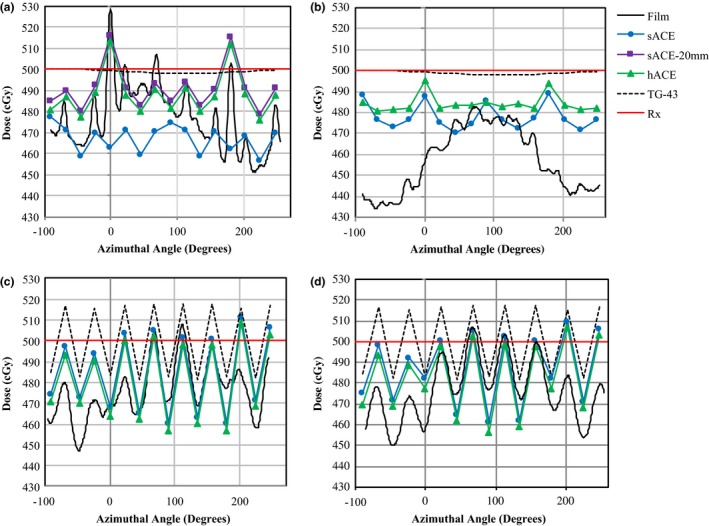
The longitudinally averaged dose to points on the surface of the MCVC applicator for the air‐in‐grooves set‐up with the central channel loaded (a), the water‐in‐grooves set‐up with the central channel loaded (b), the air‐in‐grooves set‐up with the peripheral channels loaded (c), and the water‐in‐grooves set‐up with the peripheral channels loaded (d). The dose was calculated by TG‐43, sACE, and hACE using the same dwell times corresponding to a dose of 500 cGy (TG‐43) specified to the surface of the applicator. Data points at 0° and 180° are above the two outer grooves on the surface of the applicator. The red line indicates the specified dose of 500 cGy (R_x_). The longitudinally averaged dose measured by radiochromic film on the surface of the MCVC applicator is also plotted.

Percent dose differences between TG‐43, sACE, sACE‐20 mm, and hACE calculations are given in Fig. 5 for the library model of the MCVC applicator having air in the surface grooves. The percent dose difference maps in Fig. 5 are for an axial slice through the applicator, located in the middle of the length where the dose was specified on the applicator surface. In Figs. 5(a) and 5(b), a negative dose difference indicates that sACE predicts a lower dose than TG‐43. The mean dose difference at the surface of the applicator between TG‐43 and sACE or hACE, when the peripheral channels of the MCVC are loaded, is less than 4%. This is also observed in Fig. 6, which plots the longitudinally averaged dose to points on the surface of the applicator vs. azimuthal angle when the peripheral channels are loaded. sACE and hACE predict doses within 1% of each other to the surface of the applicator, which are on average (3 ± 8)% less than the doses predicted by TG‐43. The ACE calculation margins are clearly visible in Figs. 5(a–e). In Fig. 5(a), the grid size transition from 1 mm to 2 mm is seen as the smaller square surrounding the applicator. The transition to 2 mm and 5 mm grid sizes, for hACE and sACE, respectively, is seen as the larger square in all panels of Fig. 5.

When the central channel is loaded (Figs. 5(b) and 5(c)), sACE predicts an increase in dose above the two outer grooves on the surface of the applicator. However, sACE predicts this dose increase to points that are at least 1 mm off the surface of the applicator, not directly on the surface of the applicator. hACE (Fig. 5(e)) does predict this dose increase to points directly on the surface of the applicator, as shown in Fig. 6(a), where a (6 ± 8)% increase in dose is seen above the two outer grooves compared to points not above the grooves. The sACE‐20 mm calculation (Fig. 5(c)) also predicts a (6 ± 8)% increase in dose directly above the two outer grooves. TG‐43 cannot predict this dose increase. Dose variations and mean doses to points on the surface of the applicator, as calculated by TG‐43, sACE, sACE‐20 mm, and hACE, and measured by film, are summarized in Table 3.

**Table 3:** Variations in the longitudinally averaged dose around the circumference of the MCVC applicator calculated using TG‐43, sACE, sACE‐20 mm, hACE, and measured using film. Percentages are relative to the mean dose and are given in parentheses. Expanded (k = 2) uncertainties are given

Channel/s	Material in grooves	Calculation/measurement method	Maximum Dose variation [cGy] (%)	Mean Dose (cGy)	Dose calc. time (min)	No. of transport directions (primary/residual)
Central	Air	sACE	20 ± 66 (4 ± 14)	467 ± 46	3	320/180
Central	Air	sACE‐20 mm	38 ± 68 (8 ± 14)	491 ± 50	4	320/180
Central	Air	hACE	37 ± 70 (8 ± 14)	488 ± 48	35	720/240
Central	N/A	TG‐43	2 ± 48 (0 ± 10)	499 ± 35	N/A	N/A
Central	Air	Film	77 ± 40 (16 ± 8)	477 ± 28	N/A	N/A
Central	Water	sACE	18 ± 68 (4 ± 14)	478 ± 48	3	320/180
Central	Water	hACE	15 ± 70 (3 ± 14)	484 ± 48	35	720/240
Central	Water	Film	49 ± 18 (11 ± 4)	464 ± 16	N/A	N/A
Peripheral	Air	sACE	52 ± 68 (11 ± 14)	485 ± 48	28	240/128
Peripheral	Air	hACE	52 ± 68 (11 ± 14)	482 ± 48	286	500/200
Peripheral	Air	TG‐43	36 ± 48 (7 ± 10)	500 ± 35	N/A	N/A
Peripheral	Air	Film	61 ± 38 (13 ± 8)	476 ± 30	N/A	N/A
Peripheral	Water	sACE	49 ± 68 (10 ± 14)	487 ± 48	28	240/128
Peripheral	Water	hACE	50 ± 68 (10 ± 14)	483 ± 48	286	500/200
Peripheral	Water	Film	57 ± 26 (12 ± 6)	477 ± 14	N/A	N/A

## 4 Discussion

### 4.A ACE calculation and film measurement comparison

This section will discuss and explain dosimetric phenomena observed with the MCVC applicator film measurements, and evaluate the ability of ACE to predict these phenomena. When the peripheral channels of the MCVC were loaded, a uniform oscillation in dose was measured at the surface of the applicator (Figs. 6(c) and 6(d)). This dose variation is not associated with a heterogeneity; it is an expected variation due to the proximity of the peripheral channels to the surface of the applicator not being able to create a perfectly uniform dose distribution, and is therefore predicted by TG‐43 calculations. With the peripheral channels loaded, the maximum dose variation as determined by sACE and hACE was (11 ± 14)% of the mean dose, and is within the uncertainty of the measured dose variation of (13 ± 8)% of the mean dose. TG‐43 calculations yielded a maximum dose variation of (7 ± 10)% of the mean dose.

There was a measured increase of (11 ± 8)% of the mean dose at the surface of the applicator above the two outer grooves, when they were filled with air, and the central channel was loaded. This dose increase was also observed with hACE calculations and was (6 ± 8)% of the mean dose (Fig. 6(a)). sACE did not predict a dose increase above the two outer grooves to points that are on the surface of the applicator, but did predict a dose increase of 6% at a distance of at least 1 mm off the surface of the applicator, as seen in Fig. 5(b). When the calculation was performed using sACE‐20 mm, such that the grid size at the surface of the applicator is 1 mm, a (6 ± 8)% increase in dose was seen at the surface of the applicator (Fig. 6(a)). The calculation grid size is the same for both the primary dose and scatter dose calculations. The 2 mm grid size of sACE will average the attenuation coefficients between the air and water, reducing the increase in dose at grid points right at the surface of the applicator. Additionally, dose points at a location not on the calculation grid are determined by trilinear interpolation between neighboring grid points. Therefore, depending on the alignment of the grid, the doses at the applicator surface could be averaged between doses in air and water. For vaginal vault HDR brachytherapy treatments at our center, a condom is usually placed around the applicator prior to insertion into the patient. Therefore, the applicator grooves are not filled with fluids from the patient, which would likely result in peak dose variations similar to those observed in the air‐in‐grooves film measurements. In pulsed dose rate (PDR) gynecological brachytherapy treatments, the longer treatment times require use of a perineal bar for immobilization of the applicator, and a condom is not placed around the applicator. In this case, the grooves may fill with bodily fluids from the patient causing a reduction or elimination of the peak dose variations.

**Figure 7:**
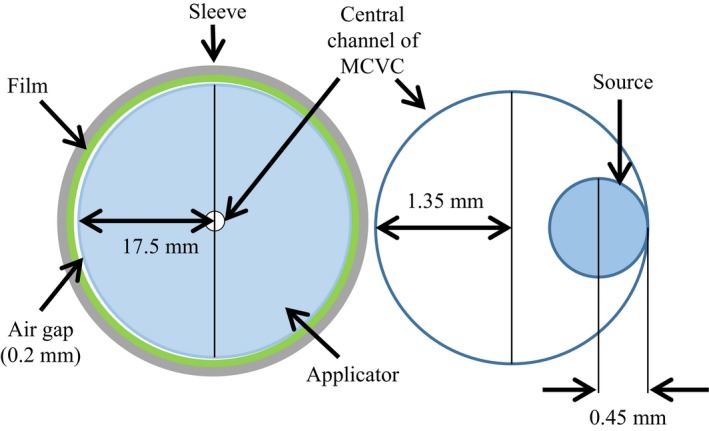
Illustration of the worst case geometry for the Ir‐192 source in the central channel of the MCVC for the film measurements. The combination of the source being at the edge of the central channel, and the 0.2 mm air gap between the applicator and film, causes a difference in dose at opposite sides of the applicator surface.

In addition to the increased dose at the grooves, a baseline dose variation was measured around the surface of the applicator. The average baseline dose variation for either water or air in the grooves was (10 ± 4)% of the mean dose. This dose variation around the circumference of the MCVC applicator is likely due to offset positioning of the source within the central channel and is not due to applicator heterogeneity. The variation was also observed when similar film measurements were performed for another vaginal cylinder containing only a central channel (Elekta, Stockholm, Sweden, part #084.354). The MCVC applicator central channel has a diameter of 2.7 mm as measured from the μCT images, while the Ir‐192 HDR v2 source has a diameter of 0.9 mm. When all dwell positions are in contact with the channel wall and the film is 0.2 mm off the surface of the applicator on the opposite side, the dose to the active layer of the film farthest from the dwell positions will be 91% of the dose to the film closest to the dwell positions (largest possible offset), which corresponds to a difference of about 9% of the mean dose. This dose difference was computed using an average IS correction, weighted with respect to the dwell times for each dwell position, to a location midway down the irradiated portion of the applicator. Fig. 7 illustrates the dimensions of the source and central channel. The library model in OcB Applicator Modeling defines a fixed source path along the center of the catheter lumen in the absence of any additional information about source positioning. Verification of the source path via imaging may be desirable when the catheter diameter is significantly larger than the source.^26^, ^27^

Lastly, all of the film measurements and ACE calculations yielded average doses 3–7% less than the doses calculated using TG‐43, but none of these differences were statistically significant.

The radiation oncologists at our clinic advise that a localized increase in dose of 11%, as observed above the two outer grooves when filled with air, would not constitute a significant clinical concern on its own. However, if the dose variation was greater than 20%, which is possible when combining the peak dose increase and baseline dose variation, it may necessitate adjustment of the plan. Another consideration that may contribute to a larger dose variation is the presence of air pockets surrounding the applicator.^28^, ^29^, ^30^ When considering TG‐43 calculations alone, it was found that 11 of 174 (6.3%) patients were underdosed by an average of 6.1% of the prescribed dose due to displacement of the vaginal mucosa by air gaps.^28^ A phantom study by Maxwell et al.^31^ found that TG‐43 slightly underestimates the dose due to the inhomogeneity caused by the presence of air – the dominant effect is a decreased dose to tissue due to increased distance from the source.

### 4.B sACE and hACE calculations

The difference between sACE and hACE calculations for the MCVC was dependent on whether the central channel or peripheral channels were loaded. As previously stated in section 4.A, the hACE calculation predicted an increase in dose above the two grooves on the surface of the applicator, whereas the sACE calculation did not due to the larger calculation grid size. In general, sACE was observed to compute lower doses than hACE, for the majority of the dose points, relative to TG‐43 (Fig. 5). The lower doses computed by sACE can be explained by the “ray effect”, which was investigated for ACE by Ma et al.^32^ In collapsed‐cone convolution algorithms, energy is transported along the axis of a cone‐shaped scatter kernel. When the radius of the kernel is larger than the grid size, too much energy is deposited along the central axis, thereby overestimating the dose to those grid points, and underestimating the dose to the majority of the grid points lying between the axes. The ray effect is less pronounced in the hACE calculation because the number of transport directions for primary scatter is 720 whereas sACE uses 320. Therefore, sACE has a tessellation triangle that covers an area over twice the size of the hACE triangle, but the grid size of sACE is exactly twice the size of hACE on the surface of the applicator, resulting in the sACE cone containing relatively more grid elements than the hACE cone. When the peripheral channels of the applicator are used, sACE and hACE have a 1 mm grid size at the surface of the applicator and predict similar doses to the surface of the applicator that are less than 1% different. The peripheral channels are also much closer to the surface of the applicator; therefore the opening area of the cone is smaller, which will reduce the ray effect. Additionally when the peripheral channels are used, and despite the hACE calculation having approximately twice the number of primary scatter transport lines than sACE, the numerous dwell positions “wash‐out” the ray effect for the sACE calculation. This occurs due to overlapping transport lines from the many dwell positions.^32^

## 5 Conclusion

Triple channel radiochromic film dosimetry was performed to verify Oncentra^®^ Brachy ACE v4.5 calculations for a multichannel vaginal cylinder applicator, and comparisons between TG‐43 and ACE dose calculations were used to evaluate the clinical significance of applicator‐heterogeneity‐induced dose variations. High accuracy ACE dose calculations were found to agree with film measurements when just the central channel of the applicator was loaded and when just the peripheral channels of the applicator were loaded. Standard accuracy ACE dose calculations did not predict an increase in dose to the applicator surface above two outer applicator grooves when they were filled with air, and TG‐43 calculations cannot predict this increase, therefore high accuracy ACE is recommended when needing to resolve similar interfaces. When the peripheral channels of the applicator were used, the agreement between standard and high accuracy ACE calculations suggests that standard accuracy is sufficient when dwell positions are located close to prescription points. The film measurements also revealed a baseline azimuthal dose variation on the applicator surface, likely due to non‐axial positioning of the Ir‐192 source in the central channel, which could not be accounted for in the calculations when using the multichannel vaginal cylinder model in OcB Applicator Modeling that assumes axial positioning of the source. Clinical use of the multichannel applicator should take the dosimetric effects reported here into consideration within the context of the particular treatment being planned.

## Conflict of Interest

The authors have no relevant conflicts of interest to disclose.

## Appendix A

### Uncertainties for Film Measurements

#### Uncertainty in the use of the derived calibration curve to determine a measured dose

This uncertainty follows directly from the methodology outlined by Morrison et al.^21^ The combined uncertainty from the measured optical density (*σ*_*OD*_), the calibration curve parameters (*σ*_*A*_ *, σ*_*B*_ *, σ*_*C*_), and the reference linac doses (*σ*_*Dref*_), gives the total uncertainty for a single dose measurement (*D*) and a single color channel (*C*1):

(A1) $$\begin{array}{cc} & \sigma_{D,C1}\left(\%\right)\\ & =\frac{100\cdot \sqrt{{\left(\frac{\partial D_{film}}{\partial OD}\right)}^{2}{\sigma_{OD}}^{2}+{\left(\frac{\partial D_{film}}{\partial A}\right)}^{2}{\sigma_{A}}^{2}+{\left(\frac{\partial D_{film}}{\partial B}\right)}^{2}{\sigma_{B}}^{2}+{\left(\frac{\partial D_{film}}{\partial C}\right)}^{2}{\sigma_{C}}^{2}+{\left(\frac{\partial D_{film}}{\partial D_{ref}}\right)}^{2}{\sigma_{Dref}}^{2}}}{D_{film}} \end{array}$$

#### Propagation of calibration curve uncertainty by weighting the dose with uncertainties associated with each color channel, and using triple channel analysis

Let *σ*_*D,C*1_, *σ*_*D,C*2,_ and *σ*_*D,C*3_ be *σ*_*i*_ for i = 1, 2, 3 = C1, C2, C3.

Let k = single color channel.

The weight (*w*) for the dose (*D*), determined by the calibration curve for each color channel, is given by Eq. (A2). The total weighted dose (*D*_*w*_) is given by Eq. (A3).

(A2) $$w_{k}\left(D\right)={\left(\frac{\sum_{i=1}^{3}\sigma_{i}\left(D\right)}{\sigma_{k}\left(D\right)}\right)}^{2}$$

(A3) $$D_{w}=\frac{\sum_{i=1}^{3}D_{i}w_{i}{\left(D\right)}^{-2}}{\sum_{i=1}^{3}w_{i}{\left(D\right)}^{-2}}$$

The uncertainty of the weighted dose is given by:

(A4) $$\sigma_{D_{w}}\left(D\right)=\frac{1}{\sum_{i=1}^{3}w_{i}{\left(D\right)}^{-2}}\cdot {\left(\sum_{i=1}^{3}w_{i}{\left(D\right)}^{-4}\sigma_{i}{\left(D\right)}^{2}{D_{i}}^{2}\right)}^{1/2}$$

The uncertainty from the triple channel analysis is given by:

(A5) σTC=13Dw∑i,j=1i≠j3|Di−Dj|

The total uncertainty for the weighted dose determined from the calibration curves and triple channel analysis is the quadrature sum of the triple channel uncertainty and weighted dose uncertainty. The total uncertainty for a single film pixel will henceforth be referred to as the type B film uncertainty:

(A6) $$\sigma_{B,Film}=\sqrt{{\sigma_{TC}}^{2}+{\sigma_{D_{w}}}^{2}}$$

#### Resulting uncertainty in average value of dose made from multiple dosimetric measurements

The type A uncertainty associated with averaging N pixels that have a standard deviation in dose *σ*_*D*_:

(A7) $$\sigma_{A,Film}=\frac{\sigma_{D}}{\sqrt{N}}$$

#### Total uncertainty for an average film dose $\overset{¯}{D}$ determined from N pixels

(A8) $$\sigma_{\overset{¯}{D},Film}=\sqrt{\sigma_{B,Film}{\left(\overset{¯}{D}\right)}^{2}+\sigma_{A,Film}{\left(\overset{¯}{D}\right)}^{2}}$$

## Appendix B

### Uncertainties for TG‐43 and ACE calculations

#### B1. Uncertainty for a single dose point

For a single dose point calculated by TG‐43 in OcB, the combined type B uncertainty is obtained from the combination of uncertainties from the air kerma strength (*S*_*k*_), the reference Monte Carlo dose estimate, and the treatment planning system interpolation. The values used here are from DeWerd et al.^23^ for high energy BT sources:

(B1) $$\sigma_{B,calc}=\sqrt{\sigma_{S_{k}}^{2}+\sigma_{MC}^{2}+\sigma_{TPS}^{2}}=\sqrt{1.5^{2}+1.6^{2}+2.6^{2}}=3.4\%$$

The uncertainty for a single dose point calculated by ACE is estimated to be 5%.^24^, ^25^

#### B2. Type A uncertainty associated with the average dose taken from N dose points with the standard deviation *σ*_*D,*calc_

(B2) $$\sigma_{A,calc}=\frac{\sigma_{D,calc}}{\sqrt{N}}$$

#### B3. Total uncertainty for an average calculated dose $\overset{¯}{D}$ determined from N dose points

(B3) $$\sigma_{\overset{¯}{D},calc}=\sqrt{\sigma_{B,calc}^{2}+\sigma_{A,calc}^{2}}$$

## Appendix C

### Uncertainty in percent dose differences between average TG‐43 and ACE calculated doses or average TG‐43 doses and average film measurements

Let *a* denote an absolute uncertainty and *r* denote a relative uncertainty.

$$\begin{array}{cc} & \Delta D\left(\%\right)=100\cdot \frac{\left({\overset{¯}{D}}_{film}\pm \sigma_{a,film}\right)-\left({\overset{¯}{D}}_{TG43}\pm \sigma_{a,TG43}\right)}{({\overset{¯}{D}}_{TG43}\pm \sigma_{a,TG43})}\\ & =\frac{100\cdot \left({\overset{¯}{D}}_{film}-{\overset{¯}{D}}_{TG43}\right)\pm \sqrt{\sigma_{a,film}^{2}+\sigma_{a,TG43}^{2}}}{\left({\overset{¯}{D}}_{TG43}\pm \sigma_{a,TG43}\right)}\\ & =\frac{100\cdot \left({\overset{¯}{D}}_{film}-{\overset{¯}{D}}_{TG43}\right)\pm \sigma_{a,diff}}{\left({\overset{¯}{D}}_{TG43}\pm \sigma_{a,TG43}\right)}\\ & =\frac{100\cdot ({\overset{¯}{D}}_{film}-{\overset{¯}{D}}_{TG43})}{{\overset{¯}{D}}_{TG43}}\pm \sqrt{\sigma_{r,diff}^{2}+\sigma_{r,TG43}^{2}}=\Delta D\left(\%\right)\pm \sigma_{r,\Delta D}\left(\%\;of\;\%\right)\\ & =\Delta D\left(\%\right)\pm \sigma_{a,\Delta D}\left(\%\right) \end{array}$$
